# Uncovering carbohydrate metabolism through a genotype-phenotype association study of 56 lactic acid bacteria genomes

**DOI:** 10.1007/s00253-019-09701-6

**Published:** 2019-03-04

**Authors:** Gemma Buron-Moles, Anna Chailyan, Igor Dolejs, Jochen Forster, Marta Hanna Mikš

**Affiliations:** 1 Carlsberg Research Laboratory, J.C. Jacobsens Gade 4, 1799 Copenhagen V, Denmark; 2 Faculty of Food Science, University of Warmia and Mazury, Plac Cieszyński 1, 10-726 Olsztyn, Poland; 3 Present Address: Glycom A/S, Kogle Allé 4, 2970 Hørsholm, Denmark

**Keywords:** Carbohydrate metabolism, Genome-wide association study, Genotype-phenotype association study, Lactic acid bacteria, Functional genomics, Microbial genomics

## Abstract

Owing to their unique potential to ferment carbohydrates, both homo- and heterofermentative lactic acid bacteria (LAB) are widely used in the food industry. Deciphering the genetic basis that determine the LAB fermentation type, and hence carbohydrate utilization, is paramount to optimize LAB industrial processes. Deep sequencing of 24 LAB species and comparison with 32 publicly available genome sequences provided a comparative data set including five major LAB genera for further analysis. Phylogenomic reconstruction confirmed *Leuconostoc* and *Pediococcus* species as independently emerging from the *Lactobacillus* genus, within one of the three phylogenetic clades identified. These clades partially grouped LABs according to their fermentation types, suggesting that some metabolic capabilities were independently acquired during LAB evolution. In order to apply a genome-wide association study (GWAS) at the multigene family level, utilization of 49 carbohydrates was also profiled for these 56 LAB species. GWAS results indicated that obligately heterofermentative species lack 1-phosphofructokinase, required for d-mannose degradation in the homofermentative pathway. Heterofermentative species were found to often contain the *araBAD* operon, involved in l-arabinose degradation, which is important for heterofermentation. Taken together, our results provide helpful insights into the genetic determinants of LAB carbohydrate metabolism, and opens for further experimental research, aiming at validating the role of these candidate genes for industrial applications.

**Electronic supplementary material:**

The online version of this article (10.1007/s00253-019-09701-6) contains supplementary material, which is available to authorized users.

## Introduction

With the increased accessibility of next-generation sequencing (NGS) and third-generation sequencing technologies, including high-throughput sequencing (HTS) and whole-genome sequencing (WGS), the number of sequenced genomes has grown significantly, from approx. 31,000 in year 2014 to more than 160,000 prokaryotic genome sequences publicly available as for today (September 2018; http://www.ncbi.nlm.nih.gov/genome/browse/). HTS technology opened a new window to study microorganism diversity on the, so far unknown, genetic scale. WGS also provided a fresh, broad insight into bacteria functionality at the genomic level, which has not been previously possible. Indeed, these emerging technologies unlocked the potential for microbial genome-wide association studies (GWAS), aiming at dissecting the genetic basis of known phenotypic traits (e.g., carbohydrate metabolism) (Dutilh et al. 2013). Understanding certain genotype-phenotype associations brings unprecedented opportunities for better utilization/exploration of technologically useful microorganisms, such as starter cultures in food technology (Wu et al. 2017), but also for the engineering of novel microbial organisms and consortia in synthetic biology applications (Freddolino et al. 2014). Bacterial evolution, epidemiology, pathogens’ traceability during disease outbreaks, pathogenesis, antibiotic resistance, rapid detection, and food safety (Chen and Shapiro 2015; Brbić et al. 2016; Klemm and Dougan 2016; Deurenberg et al. 2017; Ruppé et al. 2017), exemplify areas where sequencing and GWAS revolutionized modern microbiology.

Lactic acid bacteria (LAB) is a naturally biodiverse, well-established group of microorganisms widely used in food industry and of well-documented impact on human health (Wu et al. 2017). LABs are “generally recognized as safe” (GRAS) in the USA and given the “Qualified Presumption of Safety” (QPS) status by the European Food Safety Authority (EFSA) (Zhang and Zhang 2014). They were isolated from various ecological niches, starting from the gastrointestinal tracts of humans, animals, and insects, through plant- and meat-based materials, to soil and water (Hammes and Hertel 2009). The ability acquired by LAB to metabolize several carbohydrates provided them competitive advantages to colonize numerous ecosystems. This variety of utilized substrates leverages a few metabolic pathways, which make LAB so unique and distinctive in terms of their fermentation potential. Based on the final fermentation product(s), LAB can be divided into two groups: homo- and hetero-fermentative, the latter being subdivided into facultatively and obligately fermentative species. *Pediococcus*, *Lactococcus*, *Streptococcus*, and selected *Lactobacillus* are considered obligate homofermentative, due to their ability to ferment only hexoses almost completely to lactic acid by the Embden–Meyerhof–Parnas (EMP) pathway, while pentoses are not degraded by all homofermenters (Pessione 2012). Facultatively heterofermentative LAB species, like *Leuconostoc* and certain *Lactobacillus*, degrade hexoses to lactic acid through EMP pathway, and can also metabolize pentoses and often gluconate as they possess both aldolase and phosphoketolase (Felis and Dellaglio 2007). The obligately heterofermentative LAB cannot utilize hexoses through the EMP pathway due to the lack of glycolytic enzyme fructose-1,6-bisphosphate aldolase. Instead, they degrade hexoses by the phosphogluconate pathway, producing not only lactic acid as the end product but also significant amounts of ethanol or acetic acid and carbon dioxide (Pessione 2012).

The industrial and technological relevance of LAB strains motivated extensive genomic studies, which have provided significant insight into their metabolism, physiology, and potential for new applications (Zhang and Zhang 2014; Bosma et al. 2017). Since 2001, when the first LAB genome was published (Bolotin et al. 2001), hundreds of LAB genomes have been sequenced and are publicly available at different assembly levels (Sun et al. 2015; Zheng et al. 2015; Wu et al. 2017; Salvetti et al. 2018). It has been demonstrated that combining phenotypic data and LAB strain-specific genetic information is an effective method for the assignment of unknown functions to specific genetic loci, especially those underlying important industrial traits, interaction with the host or niche adaptation (Siezen et al. 2011; Bayjanov et al. 2013; Ceapa et al. 2015). This has provided deeper understanding of LAB’s carbohydrate metabolism and other phenotypic traits, which includes the discoveries of, e.g., arabinose and melibiose utilization genes in *Lactococcus lactis* of plant origin (Bayjanov et al. 2013), candidate genes that correlate with l-sorbose and α-methyl-d-glucoside utilization in *Lactobacillus rhamnosus* (Ceapa et al. 2015), mannose-specific adhesin in *Lactobacillus plantarum* (Pretzer et al. 2005), or specific chromosomal orthologous groups (chrOGs) identified for *Lactococcus lactis* strain (separately for subspecies *lactis* and *cremoris*) (Siezen et al. 2011). However, in many LAB genomes, a number of carbohydrate-specific genes remain to be identified.

With the aim of better understanding carbohydrate utilization and fermentation, and ultimately help to optimize industrial processes, a genotype-phenotype association study was performed in a biodiverse group of LAB. This focused on *Lactobacillus* spp. potentially harboring industrial applications. Other four genera were added for comparative purposes, namely *Lactococcus*, *Pediococcus*, *Leuconostoc*, and *Streptococcus*. The genome of 24 LAB species was sequenced and compared to 32 already available, in order to define multigene families across the 5 LAB genera. Variation in multigene family size was then evaluated for association with 49 phenotypic traits, which characterize LAB carbohydrate metabolism. This analysis identified novel multigene families involved in carbohydrate degradation, as well as their relation with the respective LAB fermentation pathways.

## Materials and methods

### Bacterial cultures and dataset

A representative set of 50 type strains of LAB was selected to cover a bio-diverse group of microorganisms with potential industrial applications, mostly with GRAS/QPS status, different fermentation capacities, and strains’ origins (Table 1). In addition, six in-house *Lactococcus* spp. isolates were also included (Carlsberg Research Laboratory, Copenhagen, Denmark). In total, 56 LAB strains have been examined comprising species from the following genera: *Lactobacillus* (*n* = 42), *Lactococcus* (*n* = 8), *Leuconostoc* (*n* = 3), *Pediococcus* (*n* = 2), and *Streptococcus* (*n* = 1). The comparative panel of LAB strains included homofermentative (*n* = 31), heterofermentative (*n* = 4), facultatively heterofermentative (*n* = 12), and obligately heterofermentative (*n* = 9) representatives. The taxonomic status of *Streptococcus salivarius* spp. *thermophilus* has been contentious. Some authors have proposed that *St*. *salivarius* spp. *thermophilus* is not a subspecies of *St*. *salivarius*, but a separate species (Facklam 2002). Hereafter, ATCC 19258 is referred to as *St*. *salivarius* spp. *thermophilus*, for consistency with the name used by the provider. Type strains were purchased from American Type Culture Collection (ATCC, Manassas, VA, USA), Leibniz-Institut DSMZ-Deutsche Sammlung von Mikroorganismen und Zellkulturen GmbH (DSMZ, Braunschweig, Germany), Statens Serum Institut (SSI, Copenhagen, Denmark), and Microbiologics (MicroBioLogics, St. Cloud, MN, USA). All isolates were maintained as frozen stocks in 50% (*w*/*w*) glycerol tubes at − 80 °C. Subsequently, the strains were activated prior to the experiments by double sub-culturing (of an aliquot of 100ul) in 9 ml of de Man Rogosa Sharp (MRS), or M17 (Oxoid, Basingstoke, Hampshire, England) supplemented with 0.5% glucose (Merck, Darmstadt, Germany), and incubated aerobically at optimal temperature for 24–72 h.

**Table 1:** List of 56 strains of lactic acid bacteria (LAB) used in this study, including their origin and genome sequence availability

#	Taxon	Strain code	Origin	^a^Metabolism	^b^GRAS/QPS status	NCBI accession
1*	*Lactococcus lactis* ssp. *lactis* 1	CRL 0001	Cheese	Ho	+	–
2*	*Lactococcus lactis* ssp. *lactis* 2	CRL 0002	Cheese	Ho	+	–
3*	*Lactococcus lactis* ssp. *lactis* 3	CRL 0003	Cheese	Ho	+	–
4*	*Lactococcus lactis* ssp. *lactis* 4	CRL 0004	Cheese	Ho	+	–
5*	*Lactococcus lactis* ssp. *lactis* 5	CRL 0005	Cheese	Ho	+	–
6*	*Lactococcus lactis* ssp. *lactis* 6	CRL 0006	Cheese	Ho	+	–
7*	*Streptococcus salivarius* ssp. *thermophilus*	ATCC 19258^T^	Unknown	Ho	+	PRJNA433425
8*	*Pediococcus pentosaceus*	ATCC33316^T^	Unknown	Ho	+	PRJNA434256
9*	*Lactococcus lactis* ssp. *lactis*	ATCC 19435^T^	Unknown	Ho	+	PRJNA434373
10*	*Lactococcus lactis* ssp. *cremoris*	ATCC 19257^T^	Unknown	Ho	+	PRJNA434374
11*	*Lactobacillus sakei* ssp. *sakei*	ATCC 15521^T^	“Moto” starter of sake	FHe	+	PRJNA434375
12*	*Lactobacillus amylolyticus*	DSM11664^T^	Acidified beer wort	Ho	+	PRJNA434376
13*	*Lactobacillus delbrueckii* ssp. *jakobsenii*	DSM 26046^T^	Malted sorghum wort, African dolo wort	Ho	+	PRJNA434378
14*	*Leuconostoc citreum*	ATCC 49370^T^	Honey dew of rye ear	He	+	PRJNA434381
15*	*Leuconostoc fallax*	ATCC 700006^T^	Sauerkraut	He	−	PRJNA434383
16*	*Lactobacillus silagei*	DSM 27022^T^	Orchardgrass (*Dactylis glomerata L*.) silage	He	−	PRJNA434387
17*	*Lactobacillus paracasei* ssp. *paracasei*	ATCC 25302^T^	Unknown	FHe	+	PRJNA434388
18*	*Lactobacillus parakefiri*	DSM 10551^T^	Kefir grain	OHe	−	PRJNA434396
19*	*Lactobacillus pentosus*	ATCC 8041^T^	Unknown	FHe	+	PRJNA434401
20*	*Lactobacillus farciminis*	ATCC 29644^T^	Sausage	Ho	+	PRJNA434405
21*	*Lactobacillus malefermentans*	ATCC 49373^T^	Beer	OHe	−	PRJNA434406
22*	*Lactobacillus buchneri*	ATCC 4005^T^	Tomato pulp	OHe	+	PRJNA434409
23*	*Lactobacillus pasteurii*	DSM 23907^T^	Beer contaminant	Ho	+	PRJNA434410
24*	*Lactobacillus hilgardii*	ATCC 8290^T^	Wine	OHe	+	PRJNA434413
25	*Lactobacillus delbrueckii* ssp. *bulgaricus*	ATCC 11842^T^	Yogurt	Ho	+	NC_008054
26	*Lactobacillus delbrueckii* ssp. *delbrueckii*	ATCC 9649^T^	Sour grain mash	Ho	+	NZ_AZCR00000000
27	*Lactobacillus delbrueckii* ssp. *lactis*	ATCC 12315^T^	Emmental cheese	Ho	+	NZ_AZDE01000001
28	*Lactobacillus helveticus*	ATCC 15009^T^	Emmental cheese	Ho	+	NZ_AZEK01000001
29	*Lactobacillus dextrinicus*	ATCC 33087^T^	Silage	Ho	+	NZ_AYYK01000004
30	*Lactobacillus mali*	ATCC 27053^T^	Apple juice from cider press	Ho	+	NZ_AYYH01000001
31	*Lactobacillus acidophilus*	ATCC 4356^T^	Gastrointestinal tract and mouth	Ho	+	NZ_AZCS01000001
32	*Pediococcus acidilactici*	ATCC 8042^T^	Unknown	Ho	+	NZ_GL397067
33	*Lactobacillus nagelii*	ATCC 700692^T^	Partially fermented wine	Ho	−	NZ_AZEV01000035
34	*Lactobacillus salivarius*	ATCC 11741^T^	Saliva	Ho	+	NZ_GG693223
35	*Lactobacillus fermentum*	ATCC 14931^T^	Fermented beets	OHe	+	NZ_GG669900
36	*Lactobacillus reuteri*	ATCC 23272^T^	Intestine of adult	OHe	+	NC_009513.1
37	*Lactobacillus sakei* ssp. *carnosus*	DSM 15831^T^	Fermented meat product	FHe	+	NZ_AZFG01000049
38	*Lactobacillus sanfranciscensis*	ATCC 27651^T^	San Francisco sourdough	OHe	+	NZ_AYYM01000001
39	*Lactobacillus vini*	DSM 20605^T^	Grape must, fermenting at high temperature	FHe	−	NZ_AYYX01000001
40	*Lactobacillus amylovorus*	ATCC 33620^T^	Cattle waste-corn fermentation	Ho	+	NZ_AZCM01000001
41	*Lactobacillus composti*	DSM 18527^T^	Composting material of distilled shochu residue	FHe	−	NZ_AZGA01000088
42	*Lactobacillus farraginis*	DSM 18382^T^	Composting material of distilled shochu residue	FHe	−	NZ_AZFY01000034
43	*Leuconostoc mesenteroides* ssp. *cremoris*	ATCC 19254^T^	Hansen’s dried starter powder	He	+	NZ_GG693383
44	*Lactobacillus uvarum*	DSM 19971^T^	Must of Bobal grape variety	Ho	−	NZ_AZEG01000001
45	*Lactobacillus oeni*	DSM 19972^T^	Bobal wine	Ho	−	NZ_AZEH01000039
46	*Lactobacillus zeae*	ATCC 15820^T^	Corn steep liquor	FHe	+	NZ_AZCT01000001
47	*Lactobacillus kefiri*	ATCC 35411^T^	efir grain	OHe	−	NZ_AYYV01000004
48	*Lactobacillus gasseri*	ATCC 33323^T^	Human	Ho	+	NC_008530.1
49	*Lactobacillus alimentarius*	ATCC 29643^T^	Marinated fish product	FHe	+	NZ_AZDQ00000000.1
50	*Lactobacillus gallinarum*	ATCC 33199^T^	Chicken crop	Ho	+	NZ_AZEL01000047
51	*Lactobacillus johnsonii*	ATCC 33200^T^	Human blood	Ho	+	NZ_GG670120
52	*Lactobacillus plantarum* ssp. *plantarum*	ATCC 14917^T^	Pickled cabbage	FHe	+	NZ_GL379762
53	*Lactobacillus casei*	ATCC 393^T^	Cheese	FHe	+	NZ_AZCO01000001
54	*Lactobacillus curvatus*	ATCC 25601^T^	Milk	FHe	+	NZ_AZDL01000001
55	*Lactobacillus brevis*	ATCC 14869^T^	Feces	OHe	+	NZ_AZCP01000001
56	*Lactobacillus coryniformis* ssp. *coryniformis*	ATCC 25602^T^	Silage	FHe	−	NZ_AZCN01000001

*Newly sequenced bacterial strains and de novo assembled genomes; ^T^ type strain

^a^LAB metabolism according to Felis and Dellaglio (2007): *Ho* homofermentative, *He* heterofementative, *FHe* facultatively heterofermentative, *OHe* obligately heterofermentative

^b^GRAS/QPS: generally recognized as safe/qualified presumption of safety

The dataset of 32 complete genome sequences of LAB strains (at least at the scaffold level) was obtained from the NCBI (The National Center for Biotechnology Information Microbial Genomes database) (http://www.ncbi.nlm.nih.gov/genome/browse/) on 15 August 2016. The remaining 24 LAB strains, for which sequences were not publicly available or their assemblies were incomplete (i.e., deposited only at the contig level), were newly sequenced using Illumina HiSeq technology.

### Phenotypic characterization

The fermentation patterns were characterized with a standardized system, API 50 CHL (BioMérieux, Marcy l’Etoile, France) consisting of 50 biochemical tests for the study of bacterial carbohydrate metabolism. API 50 CH was used in conjunction with API 50 CHL medium according to the manufacturer’s instructions. Briefly, active cultures of bacterial isolates or type strains were washed twice with sterile physiological saline (0.9% *w*/*v*, NaCl) and pellets were re-suspended in API 50 CHL medium (API systems, BioMérieux). Homogenized cells’ suspensions were transferred into 50-wells of the API 50 CH strips using sterile Pasteur pipettes. All wells were overlaid with sterile mineral oil (Sigma, St. Louis, MO, USA) to effect anaerobiosis. Strips were moistened and covered as recommended by the manufacturer and incubated at optimal temperature (30 or 37 °C). Changes in well color were monitored throughout the experiment, recorded after two, and verified after 10 days. For computing purposes, the results were translated to binary code, “one” for positive reaction, “zero” for negative results for all tested strains.

### Whole-genome sequencing and assembly

**Fig. 1:**
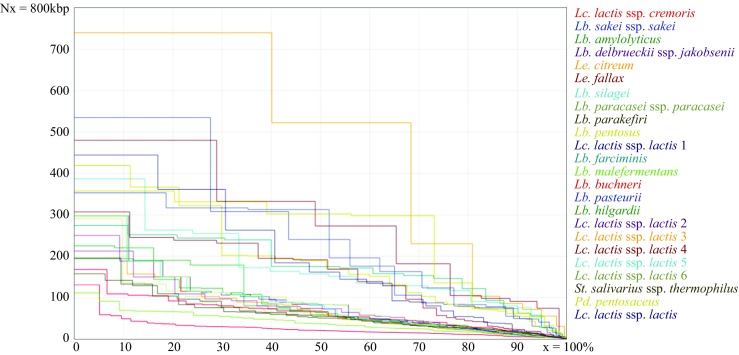
Cumulative length of contigs (Nx), as reported by QUAST. In the *x*-axis, 50% measures the N50 across the 24 new assemblies, which are color-coded as depicted in figure legend. Assemblies with higher Nx values are more contiguous

In this study, 24 LAB genomes have been sequenced using Illumina HiSeq 4000 technology. A paired-end library was constructed, and PE150 (HiSeq) sequencing targeting 100-fold coverage per each sample has been performed. Raw Illumina sequencing reads were trimmed using the CLC Genomics Workbench v 9.0 (https://www.qiagenbioinformatics.com/products/clc-genomics-workbench) with base calling error probability cutoff of *p* = 0.05 (equivalent to Phred quality score 13). Sequences shorter than 1/3 of the original read length, those containing ambiguous nucleotides, and all adapter sequences were removed. The bacterial genomes were de novo assembled using Abyss assembler (Simpson et al. 2009). For each bacterium, 12 assemblies were constructed using different *K*-*mers* (20, 32, 40, 50, 60, 65, 70, 75, 80, 85, 90, 96). Subsequently, contigs assembled for each LAB strain were evaluated with in-house scripts available upon request, retaining the best assembly for each strain, according to a number of criteria calculated by QUAST (Gurevich et al. 2013). The assembly having the lowest overall rank, calculated as the sum of the individual ranks for N50, genome length, and total number of contigs, was selected as the best. The overall report on N50 of the best assembly for each bacterium is shown in Fig. 1.

### Nucleotide sequence accession numbers

The newly sequenced genomes of 18 LAB strains, together with the corresponding gene annotations, were deposited in the GenBank database with the accession codes listed in Table 1.

### Gene prediction and functional annotation

The genome sequences were annotated in the following steps. Firstly, ab initio gene prediction of protein-coding genes for newly sequenced LAB genomes was performed using Prodigal (ver.2.6.1) software (Hyatt et al. 2010). The gff files together with protein sequence predictions were saved for each bacterium and were added to the remaining 32 bacteria for which the protein sequences have been downloaded from NCBI. The identified proteins were searched against UniFam_Prok database (159,895 families) using HMMER (ver.3.1) software (Chai et al. 2014). Top matches of proteins passing the whole-sequence *E* value cutoff (< 1e-4) and coverage cutoff (aligned regions cover at least 50% of both the matched HMM and the query protein) were selected. Proteins were then annotated with the function of their best-matched UniFam families. For each genome, the proportion of gene ontology (GO) terms was provided by the CateGOrizer software (ver.3.218) (Hu et al. 2008), according to GO_slim2 annotations and assuming the “consolidated single occurrences count” option.

### Inference of orthologous gene groups

The proteins of the 56 bacteria were clustered into orthologous groups (OGs) using OrthoFinder (Emms and Kelly 2015). First, all-versus-all searches were performed using the BLASTP algorithm (Altschul et al. 1990), with an *E* value threshold of 10^−3^. This relaxed cutoff avoids discarding putative good hits for very short sequences. At the second step, all-vs-all BLAST hits were modeled for each pairwise comparison between species, revealing and removing the gene similarity dependency on gene length and phylogenetic distance. Subsequently, the information from the reciprocal best length-normalized hit (RBNH) was used to define the lowest sequence similarity that delimits putative homologous genes. On the next step, the putative homologous gene-pairs were identified and connected in the orthogroup graph with weights given by the normalized BLAST bit scores. As final OrthoFinder step, genes were clustered into orthogroups using Markov cluster algorithm (MCL).

### Phylogenetic tree

In order to build the phylogenetic tree, 219 ortholog groups were defined by OrthoFinder (Emms and Kelly 2015) as containing a single-copy in each of the 56 LAB species (i.e., 1:1 orthologs). For each of the 219 ortholog groups, corresponding protein sequences were aligned, using MAFFT v7.310 with default parameters (Katoh and Standley 2013). The resulting 219 alignments were concatenated, yielding a multiple alignment with 74,379 positions in total. RAxML v8.2.10 (Stamatakis 2014) automatically identified LG as best-fit model for protein evolution, prior to inferring the maximum likelihood (ML) phylogenetic tree. A hundred bootstrap replicates were run to assess node support. The tree was manually rooted, assuming *St*. *salivarius* ssp. *thermophilus* and *Lc*. *lactis* group as outgroup species, in line with their basal phylogenetic placement inferred by previous phylogenetic analyses (Makarova et al. 2006; Makarova and Koonin 2007; Salvetti et al. 2013; Sun et al. 2015).

### Association of phenotypic data to genomic data

In order to carry out the association study, two data matrices were generated. Matrix A contained the protein count for each of the 56 species, in each of the 5932 orthogroups. Matrix B contained categorical phenotypes on 49 carbohydrate substrate utilization, for each of the 56 species. For each of these substrates, the probability that an orthogroup is associated with its metabolization was estimated through a non-parametric Wilcoxon rank sum test (Whitley and Ball 2002); i.e., assessing whether the number of genes within the focal orthogroup differed between species able and unable to metabolize the substrate. This amounted to a total of 290,668 statistical tests, which were adjusted for multiple testing using two different methods: Bonferroni (Bland and Altman 1995) and false discovery rate (FDR) (Benjamini and Hochberg 1995), at two different thresholds, 0.05 and 0.01.

The R packages “gplots” (Warnes et al. 2016), “devtools” (Hadley et al. 2017), “RcolorBrewer” (Neuwirth 2014), together with a customized heatmap.3 source code (Zhao et al. 2015) for hierarchical clustering based on Euclidean distance and a complete-linkage metric, have been used for results visualization (www.R-project.org). The functional annotations generated with UniFam_Prok were incorporated into the final heatmap, thus facilitating the biological interpretation of the results.

## Results

### Genome sequencing and assembly

In this study, the 56 LAB genomes were compared, of which 32 were obtained from NCBI database on 15th August 2016. For the remaining 24 LAB strains, whole-genome sequencing (WGS) and de novo assembly were performed, including representatives of the following species: *Lactobacillus* (*Lb*; *n* = 12), *Lactococcus* (*Lc*; *n* = 8), *Leuconostoc* (*Le*; *n* = 2), *Pediococcus* (*Pd*; *n* = 1), and *Streptococcus* (*St*; *n* = 1) (Table 1). The sequences of 6 strains (out of 24) were not released owing to commercial reasons. More importantly, among these 24 newly sequenced genomes, 2 type strains, *St*. *salivarius* ssp. *thermophilus* (DSM 20617) and *Le*. *citreum* (DSM 5577), were unavailable at NCBI at the time of manuscript preparation (Table 1). Finally, these 24 sequenced genomes also included 16 strains for which the assemblies were available, however, fragmented in multiple contigs.

After processing the HiSeq 4000 sequencing reads, de novo assemblies (*n* = 24) amounted to total lengths between 1.6 Mb for *Lb*. *amylolyticus* and 3.7 Mb for *Lb*. *pentosus*. Strains with greater genome sizes exhibited elevated GC content, a trend only broken by the genomes with most extreme GC contents, namely the two *Lc*. *lactis* (< 36.02%), *Lb*. *farciminis* (36.4%) and *Lb*. *delbrueckii* ssp. *jakobsenii* (50.1%) (Supplementary Table S1). The total number of contigs ranged from 10 for *Le*. *citreum* to 208 for *Lc*. *lactis* ssp. *cremoris*, showing the maximum and minimum N50 values of 522.10 kb and 20.25 kb, respectively (Fig. 1). The fraction of missing nucleotides was limited (< 0.02%).

According to these summary statistics (Supplementary Table S1), the assembly of the 16 re-sequenced LAB strains substantially improved, having now less and often longer contigs. For 6 strains (out of 16), the N50 remained unchanged, whereas it was considerably increased for the other 10 genomes, up to 3.93-fold in *Lb*. *pentosus*. Greater N50 was reflected in an increased contiguity in the respective assemblies. Concomitantly, the total number of contigs was reduced for all 16 strains, especially for the *Lb*. *parakefiri* assembly, which was fragmented in 506 contigs and here is represented by only 101 (Supplementary Table S1). Altogether, this genomic set not only constitutes a substantial extension and improvement to the LAB genomes, but has also been consistently generated with the same methodology, minimizing potential biases, and representing therefore a suitable data set for comparative genomic studies.

### Gene prediction and functional annotation

Ab initio gene prediction was conducted for the 24 new genome assemblies, yielding a minimum and maximum of protein-coding genes between 1648 in *Le*. *fallax* and 3366 in *Lb*. *pentosus*. This range is comparable to that observed for the 32 genome assemblies downloaded from NCBI, which ranges from 1175 for *Lb*. *sanfranciscensis* to 3196 in *Lb*. *composti*. Regarding the 16 re-sequenced LAB strains for which the assembly was improved, the number of annotated genes increased, as expected (Supplementary Table S1). Indeed, genes previously disrupted in different contigs were challenging to identify, but were discovered in this study due to more contiguous assemblies. Gene mis-identification due to assembly fragmentation is expected to be particularly important in LAB genomes, since these are generally compact, with approximately one gene per kb (Supplementary Table S1).

Leveraging UniFam functional annotations, gene ontology (GO) terms were assigned to the clear majority of protein-coding genes identified. This percentage ranged from 57% in *St*. *salivarius* ssp. *thermophilus* to 82% in *Lb*. *oeni*, an averaged 70% across 56 LAB strains. Each functionally-annotated gene was characterized by a mean of 3.7 GO terms. Within each LAB strain, the distribution of GO terms was similar, as reflected by the pie charts of four species that exhibit similar GO proportions despite belonging to two different phylogenetic clades, and having different fermentation types (Supplementary Fig. S1). Among the GO terms present in all 56 LAB strains, catabolism and carbohydrate metabolism were the two functional categories showing the highest coefficient of variation (1.0258 and 1.0231, respectively). Interestingly, this indicates that the relative abundance of genes regulating carbohydrate metabolism substantially differs across the 56 LAB species.

### Ortholog groups of genes

**Fig. 2:**
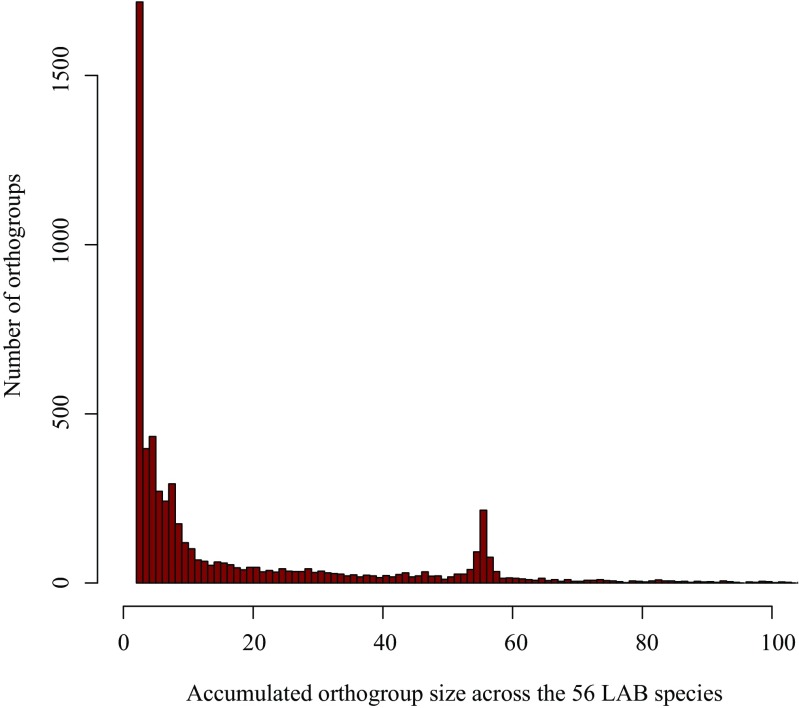
Histogram summarizing the cumulative number of genes within orthogroups, across the 56 LAB strains. For example, there are more than 1500 orthogroups (out of 5932) including 2 genes across the 56 LAB, implying either 2 genes in 1 species, or 1 gene in 2 species. Note the histogram secondarily peaks at 56, an excess reflecting the presence of the “core genome”. This is, orthogroups with a single gene copy per strain, thereby 56 in total per orthogroup

Across the 56 LAB genomes, 5932 multigene families have been identified, encompassing the 96.2% of the 123,255 predicted genes. Only 4700 (3.8%) of the total genes were not assigned to any multigene family, supporting consistent gene predictions across the 56 LAB genomes. *St*. *salivarius* ssp. *thermophilus* showed the lowest fraction of genes (82.9%) assigned to multigene families. For the remaining species, the proportion was 91.7% or above, even reaching 100% for the species *Lc*. *lactis* ssp. *lactis* 6. There were 315 multigene families identified in all 56 LAB species, of which 219 contained exactly a single gene copy per species. The results are presented in the histogram (Fig. 2) summarizing the size of the ortholog groups, which peaks at 56 genes. This peak is in line with some ortholog groups conserved as single copy in the 56 LAB genomes; i.e., each species has a single gene in each family, reflecting important genes that cannot be lost, known as the core genome (Supplementary Table S2). These 219 families represent, therefore, a minimal core genome, and reveal high turnover of gene gains and losses for the rest of the families. For example, in OG0000028, corresponding to a transposase multigene family, the number of genes ranges from 0 in the majority of species to 73 in *St*. *salivarius* ssp. *thermophilus* type strain examined in this study.

### Phylogenetic tree

**Fig. 3:**
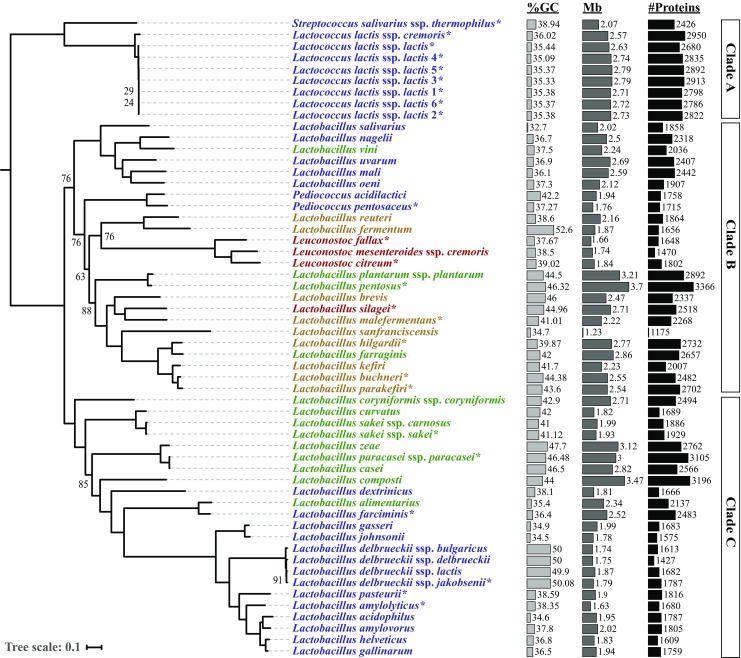
Phylogenetic tree based on the concatenate of 219 proteins from 56 LAB strains, including 24 de novo sequenced (*). LAB strains were color-coded according to Felis and Dellaglio (2007), by fermentation end-product (historically, type of fermentation): homofermentative (blue), heterofermentative (red), facultatively heterofermentative (green), and obligately heterofermentative (brown). Tree scale is given in amino acid substitutions per site. Only bootstrap values lower than 100 are shown. The GC content (%GC), genome size (Mb), and the number of predicted proteins (#Proteins) are presented as barplots. Text boxes on the right delineate the three major clades (A, B, C)

The phylogenetic relationship between the 56 LAB strains was inferred by maximum Likelihood (ML), from the concatenated alignments of the 219 proteins that constitute their core gene set (Fig. 3). Hence, branch lengths represent the number of amino acid substitutions per site. The strains of both *St*. *salivarius* ssp. *thermophilus* and *Lc*. *lactis* group were considered as outgroups, in order to root the tree. All major nodes in the tree are well-supported by high bootstrap values, demonstrating that intergroup relationships are reflected accurately.

The phylogeny clusters LAB according to their genera, separating *Leuconostoc* and *Pediococcus* within *Lactobacillus* diversity. These genera embedded in three major phylogenetic clades, namely (A), (B), and (C) (Fig. 3). Clade A corresponds to the species forced to be outgroup, which includes strains of the *Lc*. *lactis* group and *St*. *salivarius* ssp. *thermophilus*. Their basal phylogenetic placement as outgroup is robustly supported by previous studies (Makarova et al. 2006; Makarova and Koonin 2007; Salvetti et al. 2013; Sun et al. 2015). Clade B contains all possible metabolic profiles, and is comprised of three different genera, with *Pediococcus* and *Leuconostoc* emerging as two independent groups within *Lactobacillus* species. Finally, clade C only contains facultatively heterofermentative and homofermentative strains (Fig. 3).

With a few exceptions, these major phylogenetic clades do not show clear differences, in terms of distinctive GC content, genome size, or number of proteins (Fig. 3). The highest GC content was observed in the obligately heterofermentative species *Lb*. *fermentum* (52.6%), and in the clade formed by homofermentative species *Lb*. *delbrueckii* ssp. *jakobsenii*, *bulgaricus*, *delbrueckii*, and *lactis* (approx. 50% each). Another group with high GC content is the one formed by the facultatively heterofermentative species *Lb*. *zeae*, *Lb*. *casei*, and *Lb*. *paracasei* ssp. *paracasei* (47.7%, 46.5%, and 46.5%, respectively). Notably, the last two groups belong to the major clade C. The group with the highest genome size is the one formed by the facultatively heterofermentative species *Lb*. *pentosus* (3.7 Mb) and *Lb*. *plantarum* ssp. *plantarum* (3.2 Mb), followed by the facultatively heterofermentative species *Lb*. *composti* (3.5 Mb), *Lb*. *zeae* (3.1 Mb), and *Lb*. *paracasei* ssp. *paracasei* (3.0 Mb). The two strains with larger genomes *Lb*. *pentosus* (3.7 Mb) and *Lb*. *composti* (3.5 Mb), both facultatively heterofermentative species, also have the highest number of predicted protein-coding genes (3366 and 3196, respectively) (Fig. 3).

Mapping the carbohydrate fermentation patterns of the 56 LAB strains revealed that some phylogenetically-related species showed diverse fermentative capabilities. More specifically, four pairs of closely clustered LAB species: *Lb*. *nagelii*-*Lb*. *vini*; *Lb*. *silagei*-*Lb*. *malefermentans*; *Lb*. *hilgardii*-*Lb*. *farraginis*; *Lb*. *alimentarius*-*Lb*. *farciminis*, are known to form different end products of lactic acid metabolic pathway (Felis and Dellaglio 2007). For example, even though *Lb*. *alimentarius* and *Lb*. *farciminis* showed relatively high sequence similarity, currently *Lb*. *alimentarius* is classified as facultatively heterofermentative, while *Lb*. *farciminis* as homofermentative. Similarly, the homofermentative LAB *Lb*. *nagelii* clustered in the phylogenetic tree with the facultatively heterofermentative *Lb*. *vini*.

### Genotype-phenotype association study of LAB

#### Phenotypic characterization of LAB

**Fig. 4:**
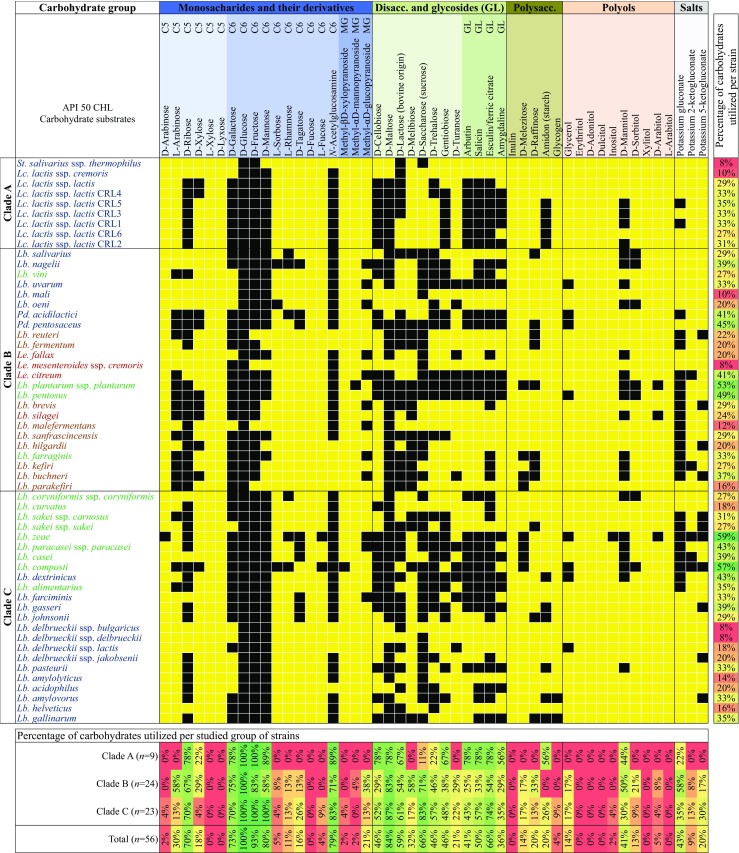
Heatmap generated based on 49 carbohydrate fermentation profiles of 56 lactic acid bacteria (LAB) strains. All carbohydrate substrates (API 50 CHL) were categorized and grouped into monosaccharides and their derivatives, disaccharides and glycosides (GL), polysaccharides, polyols, and salts. LAB’s capacity to metabolize the corresponding carbohydrate is represented as positive (black) or lack of fermentation (yellow). LAB were color-coded representing different fermentation patterns (type), according to Felis and Dellaglio (2007): homofermentative (blue), heterofermentative (red), facultatively heterofermentative (green), and obligately heterofermentative (brown). The LAB strains clustering (clades A–C) corresponds to the phylogenetic tree in Fig. 3. Numbers on the right represent the percentage of carbohydrates utilized per strain, while numbers on the bottom the percentage of carbohydrates utilized per studied group of strains. Gradient scale 0% (red)-100% (green)

The capability of 56 LAB strains to metabolize 49 carbohydrate substrates (using API 50 CHL biochemical assay) has been evaluated. The fermentation profiles of the carbohydrates demonstrated significant phenotypic diversity among tested LAB. For descriptive purposes, LAB species are hereafter defined through their representative type strains. In this regard, it is worth cautioning that some degree of strain-to-strain variation could exist within the same species (Siezen et al. 2010; Bayjanov et al. 2013; Smokvina et al. 2013; Ceapa et al. 2015), as reflected by the phenotypic profiles of *Lc*. *lactis* ssp. *lactis* strains (Fig. 4). In addition, 49 carbohydrate substrates have been grouped into monosaccharides, disaccharides, polysaccharides, polyols, and salts (Fig. 4).

Monosaccharides are the most basic, fundamental units and can be classified according to their number of carbon atoms. Among common carbon sources classified as hexoses (C6), d-glucose is the only that can be degraded by all the 56 strains. Similarly, with a few exceptions in each phylogenetic clade, the LAB studied can metabolize d-galactose, d-fructose, and d-mannose. Interestingly, all strains in clades A and C were able to degrade d-fructose. Other hexoses such as d-tagatose, d-fucose, and l-fucose can be degraded by less LAB strains, if any. For example, d-tagatose, a stereoisomer of fructose (ketohexose), can only be degraded by a few LAB members of the clades B and C, basically *Lactobacillus* and *Pediococcus* strains. While only *Lb*. *zeae* and *Lb*. *composti* strains out of the 56 can degrade l-fucose, d-fucose cannot be degraded by any of our LAB studied (Fig. 4). Among the carbon sources classified as pentoses (C5), d-arabinose, l-arabinose, d-ribose, d-xylose, and l-xylose are included. Only *Lb*. *zeae* can degrade d-arabinose, whereas different members in clades B and C, especially in clade B, were able to metabolize l-arabinose. Other pentoses such as d-ribose and d-xylose cannot be degraded by *Leuconostoc* strains. As l-xylose, d-lyxose, a stereoisomer of ribose (aldopentose), cannot be metabolized by any of the LAB tested in this study (Fig. 4).

Calculating the percentage of monosaccharides showed that none of the strains of clade A degraded l-arabinose, l-sorbose, l-rhamnose, d-tagatose, and methyl-αd-glucopyranoside, in contrast to clades B and C, where these monosaccharides are degraded by several strains (Fig. 4).

Disaccharides–carbohydrates composed of two monosaccharides joined by a glycosidic bond, such as d-maltose, d-lactose, and d-saccharose (sucrose), are common energy sources for bacterial cells. Almost all strains were able to ferment d-maltose, and those are distributed through the three major phylogenetic clades (Fig. 4). In general, all the strains can ferment d-lactose and d-saccharose with some exceptions. For example, *Leuconostoc* cannot degrade d-lactose and *Lactococcus* strains cannot utilize d-saccharose. Other disaccharides found in API strips were amygdalin, arbutin, esculin/ferric citrate, salicin, d-cellobiose, d-melibiose, and d-trehalose. The utilization of these disaccharides implies the presence of various hydrolytic enzymes that enable them to break down the substrate, such as β-glucosidase (EC 3.2.1.21) for salicin, cellobiose and melibiose or β-amylase (EC 3.2.1.28) for trehalose.

The percentage of disaccharides utilized per clade showed that d-melibiose and d-turanose are degraded by some strains of clades B and C, but by none of clade A (Fig. 4).

Polysaccharides are carbohydrates composed of at least three monosaccharide units (e.g., d-melezitose, d-raffinose) or their longer linear or branched polymers (e.g., inulin or starch and glycogen, respectively) bound with glycosidic linkages. The trisaccharide d-melezitose can be fermented by a few LAB of the clades B and C, basically *Lactobacillus* strains. In clade B, this corresponds to *Lb*. *plantarum* ssp. *plantarum*, *Lb*. *farraginis*, *Lb*. *buchneri*, and *Lb parakefiri*, whereas in clade C to *Lb*. *zeae*, *Lb*. *paracasei* ssp. *paracasei*, *Lb*. *casei*, and *Lb*. *composti* (Fig. 4). The utilization of melezitose implies, for example, the presence of the hydrolytic enzymes β-fructofuranosidase (EC 3.2.1.26) and α-glucosidase (EC 3.2.1.20) to break down the substrate. Trisaccharide d-raffinose, composed of galactose, glucose, and fructose, was utilized by three strains of clade C, and even more strains of clade B, including *Pediococcus pentosaceus* and *Lactobacillus* strains. Raffinose can be hydrolyzed to d-galactose and sucrose by the enzyme α-galactosidase (EC 3.2.1.22), an enzyme not found in the human digestive tract. Starch is a polysaccharide formed by a large number of glucose units. It is interesting to note that species of clade B, including *Leuconostoc*, *Pediococcus*, and *Lactobacillus* strains, were not able to metabolize starch. The species that can ferment starch included *Lc*. *lactis* ssp. *lactis* 1–3, 5, and 6 (clade A), and *Lb*. *dextrinicus*, *Lb*. *gasseri*, *Lb*. *johnsonii*, *Lb*. *pasteurii*, *Lb*. *amylovorus*, and *Lb*. *gallinarum* (clade C). The enzyme responsible for hydrolysis of starch molecules yielding glucose and maltose is α-amylase (E.C.3.2.1.1). Other two polysaccharides shown in Fig. 4 were inulin and glycogen. None of tested strains were able to utilize inulin, but *Lb*. *amylovorus* and *Lb*. *gallinarum* were capable of fermenting glycogen.

A polyol is an alcohol containing three or more hydroxyl groups, and examples include glycerol, erythritol, d-adonitol, dulcitol, inositol, d-mannitol, d-sorbitol, xylitol, d-arabitol, and l-arabitol. The strains that were able to ferment glycerol belonged to *Lb*. *uvarum*, *Pd*. *acidilactici*, *Pd*. *pentosaceus*, *Lb*. *pentosus*, *Lb*. *zeae*, *Lb*. *composti*, *Lb*. *dextrinicus*, and *Lb*. *delbrueckii* ssp. *lactis*. No strain in clade A was capable of degrading glycerol, commonly used as carbon source in biotechnology. Among all the polyols, d-mannitol is the sugar alcohol most widely fermented by LAB members of each clade, followed by d-sorbitol with the exception of members in clade A, which cannot degrade d-sorbitol. On the other hand, while three strains were capable of fermenting d-arabitol (*Lb*. *plantarum* ssp. *plantarum*, *Lb*. *silagei*, and *Lb*. *zeae*), only *Lb*. *zeae* was able to degrade inositol. As for the remainder of the polyols, 56 analyzed LAB strains cannot ferment erythritol, d-adonitol, dulcitol, xylitol, and l-arabitol (Fig. 4).

The salts characterized included potassium gluconate, potassium 2-ketogluconate, and potassium 5-ketogluconate (Fig. 4). Potassium gluconate is the potassium salt of gluconic acid, which is obtained from glucose by fermentation and subsequent neutralization with a potassium source. Of the three salts characterized, potassium gluconate was the one most widely fermented by LAB members of each clade, followed by potassium 5-ketogluconate with the exception of members in clade A, which cannot metabolize this salt. Finally, five strains were capable of metabolizing potassium 2-ketogluconate being *Le*. *citreum* and *Lb*. *kefiri* (clade B), *Lb*. *zeae*, *Lb*. *casei*, and *Lb*. *composti* (clade C) (Fig. 4).

#### Association of phenotypic data to genomic data

The genotype-phenotype association analysis was performed to identify significant associations between gene family expansions/contractions in the 56 LAB strains in relation to their carbohydrate metabolism, based on three different correction methods for multiple testing, namely Bonferroni (*p* < 0.05), false discovery rate (FDR) (*p* < 0.01), and FDR (*p* < 0.05).

**Fig. 5:**
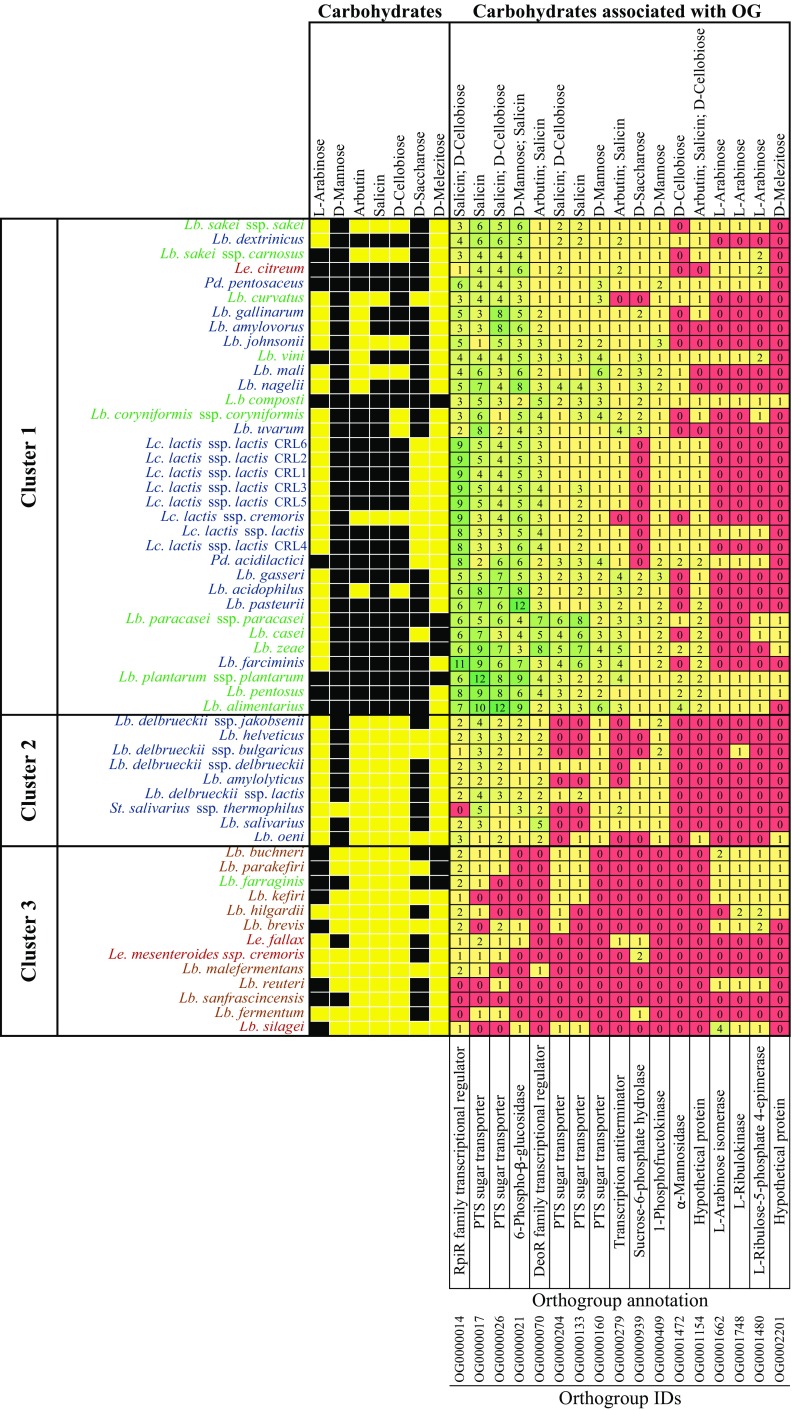
Heatmap representing significant genotype-phenotype associations. The 17 orthogroups significantly associated with the metabolism of seven carbohydrates, as identified after Bonferroni correction (*p* < 0.05), is shown. LAB species were color-coded as homofermentative (blue), heterofermentative (red), facultatively heterofermentative (green), and obligately heterofermentative (brown). Phenotype information is represented in black/yellow color scheme, based on the LAB capacity to metabolize the corresponding carbohydrate: black (positive) and yellow (negative). Gene content of the 56 LAB strains is represented in red/green color scheme, with green color indicating a greater number of genes for a given orthogroup

The results for Bonferroni correction, represented as a heatmap in Fig. 5, showed significant associations of 17 ortholog groups with 7 phenotypic traits related to carbohydrate metabolism. In Fig. 5, the 56 LAB strains clustered according to similarities in their carbohydrate metabolism, partially recovering the phylogenetic clades in Fig. 3. This implies that some of these metabolic traits evolved along the diversification of the 56 LAB species. Three clusters were observed, but not clearly separated based on fermentation type. While the first cluster comprises from *Lb*. *sakei* ssp. *sakei* to *Lb*. *alimentarius*, and excludes obligately heterofermentative species, the second cluster only includes homofermentative species, revealing similarities in their carbohydrate metabolism. By contrast, in the third cluster, the heterofermentative species grouped together, including facultatively and obligately, showing similar fermentation profiles. Among the strains of all the homofermentative species, we observed a general lack of genes belonging to multigene families associated with the phenotypes l-arabinose and d-melezitose. Similarly, the strains of obligately heterofermentative species were depleted of genes belonging to multigene families associated with the phenotypes d-mannose, arbutin, and salicin (Fig. 5).

The *Lactococcus* genus clustered together, as these strains basically showed the same carbohydrate profile, with a few exceptions. By contrast, species of the *Pediococcus* genus did not cluster according to their metabolic profiles. The main difference is that *Pd*. *acidilactici* did not metabolize d-saccharose, in contrast to *Pd*. *pentosaceus*. In addition, the orthogroup OG0000939, significantly associated with the metabolism of d-saccharose (*p* value = 6.19e-03), was present only in *Pd*. *pentosaceus*. Similarly, *Leuconostoc* species did not cluster together, since *Le*. *citreum* showed a slightly different metabolic profile compared to *Le*. *fallax* and *Le*. *mesenteroides* ssp. *cremoris*. In particular, *Le*. *fallax* and *Le*. *mesenteroides* ssp. *cremoris* have lost their ability to metabolize arbutin, salicin, d-cellobiose, d-mannose, and l-arabinose. The orthogroups significantly associated with these metabolisms were OG0000070, OG0000204, OG0000133, OG0000160, OG0000409, OG0001662, OG0001748, and OG0001480 (Fig. 5).

Differences between the three clusters defined by the heatmap lied down mainly in the ability of the strains to metabolize arbutin, salicin, and d-cellobiose (Fig. 5). Strains belonging to cluster 1 were often able to metabolize these three carbohydrates, in contrast to those belonging to clusters 2 and 3. Several orthogroups showed concomitant variations in the number of family members. For example, the number of genes in OG0000014 was at least 11 among strains that metabolize salicin and d-cellobiose, while merely 1 for the remaining. In fact, *St*. *salivarius* ssp. *thermophilus*, *Lb*. *reuteri*, *Lb*. *sanfranciscensis*, and *Lb*. *fermentum* did not metabolize salicin and d-cellobiose, and have thus no genes belonging to OG0000014. Genes within this orthogroup were functionally annotated as RpiR family transcriptional regulator. Likewise, orthogroup OG0000070 was significantly associated with the metabolism of arbutin (*p* value = 0.03) and salicin (*p* value = 0.01), and annotated as DeoR family transcriptional regulator. *Lb*. *zeae* showed the largest number of genes in OG0000070, with eight members. Interestingly, all strains in clusters 2 and 3 lost their ability of fermenting arbutin and salicin; however, only heterofermentative species in cluster 3 have almost no genes belonging to this orthogroup. Another orthogroup significantly associated with the metabolism of arbutin and salicin was OG0000279 (*p* values = 0.02 and 7.35e-04, respectively), which has been annotated as transcription anti-terminator. Unlike in cluster 2 and 3, all strains comprising cluster one had at least one gene in OG0000279, except *Lb*. *curvatus* and *Lc*. *lactis* ssp. *cremoris*, where this multigene family was absent (Fig. 5).

Orthogroups OG0000017, OG0000026, OG0000204, OG0000133, and OG0000160 were also associated with the metabolism of salicin, d-cellobiose, and d-mannose. These five orthogroups contained at least one gene present in all the LAB of cluster 1, with OG0000017 and OG0000026 harboring the greatest number of genes. In fact, *Lb*. *plantarum* ssp. *plantarum* and *Lb*. *alimentarius* contained 12 members in orthogroups OG0000017 and OG0000026, respectively. Regarding cluster 2, these orthogroups were mostly present in homofermentative species, in comparison to heterofermentative strains in cluster 3, where these families were nearly absent, or even absent for orthogroup OG0000160. While the orthogroup OG0000160 was specifically associated with the d-mannose metabolism, OG0000017 and OG0000026 were associated with salicin, and salicin and d-cellobiose, respectively. Genes belonging to all these orthogroups were functionally annotated as phosphotransferase (PTS) sugar transporters (Fig. 5). The capacity of utilizing salicin, d-cellobiose and d-mannose indicates that the genome of these LAB encode for proteins acting as specific transporters, such as PTS-salicin, PTS-cellobiose, and PTS-mannose.

The multigene family annotated as 6-phospho-β-glucosidase (OG0000021) was significantly associated with the ability to utilize d-mannose and salicin, as sucrose-6-phosphate hydrolase (OG0000939) was with d-saccharose, 1-phosphofructokinase (OG0000409) with d-mannose, and α-mannosidase (OG0001472) with d-cellobiose (Fig. 5). All LAB in cluster 1 and 2 have different copies of the 6-phospho-β-glucosidase and 1-phosphofructokinase genes. By contrast, heterofermentative LAB strains in cluster 3 have lost their 6-phospho-β-glucosidase and 1-phosphofructokinase genes, excepting *Lb*. *brevis*, *Le*. *fallax*, and *Lb*. *silagei*, which retain one gene of the 6-phospho-β-glucosidase. In cluster 1, the sucrose-6-phosphate hydrolase multigene family was only absent in *Lb*. *curvatus*, *Lc*. *lactis* group, and *Pd*. *acidilactici* strains, whereas in cluster 2 and 3, different homofermentative and heterofermentative LAB species lost their ability to ferment d-saccharose, as well as their associated genes (OG0000939). All homofermentative and heterofermentative strains in clusters 2 and 3 lost the ability to ferment d-cellobiose, as well as the α-mannosidase genes (OG0001472), which are not found in any member of these clusters (Fig. 5).

Other statistically significant genotype-phenotype association were identified between l-arabinose and the following multigene families OG0001662, OG0001748, and OG0001480, which were annotated as l-arabinose isomerase (EC 5.3.1.4; *p* value = 1.41e-05), l-ribulokinase (EC 2.7.1.16; *p* value = 5.42e-04), and l-ribulose-5-phosphate 4-epimerase (EC 5.1.3.4, *p* value 4.63e-03). As for the multigene family l-arabinose isomerase (OG0001662), *Lb*. *silagei* that metabolize l-arabinose contained four gene copies, *Lb*. *buchneri* contained two, and the rest contained one gene each. In general, correlation between the presence of l-arabinose isomerase, l-ribulokinase, and l-ribulose-5-phosphate 4-epimerase genes and the fermentation type was identified, whereby heterofermentative strains showed a greater number of these genes than the homofermentative ones. Importantly, species metabolizing and non-metabolizing l-arabinose are interspersed among *Lactobacillus* and *Lactococcus*, as well as *Leuconostoc* genus. This confirms that this genotype-phenotype association is not spuriously reflecting the underlying phylogenetic relationship, and reveals that multiple species underwent independent changes in the arabinose metabolism. Remarkably, and in contrast to the majority of carbohydrates, the isomer l-arabinose is more common than d-arabinose in nature. Collectively, this suggests that only those LAB strains able to transform l- into d-arabinose, due to the expansion of these three multigene families, are also able to subsequently metabolize d-arabinose.

Moreover, the genotype-phenotype association analysis revealed that two orthogroups predicted as unknown hypothetical proteins (functionally unannotated families; OG0001154 and OG0002201) were involved in a series of carbohydrate metabolic functions (Fig. 5). The orthogroup OG0001154 was significantly associated with the utilization of arbutin, salicin, and d-cellobiose. In cluster 1, only a few strains lost the genes of this orthogroup, whereas it was absent among homofermentative and heterofermentative species of clusters 2 and 3, with the exception of *Lb*. *oeni*, which contained one gene only. The other orthogroup of unidentified function was OG0002201, and it was significantly associated with d-melezitose metabolism. With the exception of *Lb*. *oeni* (homofermentative), only heterofermentative strains contained OG0002201 genes.

The results above proved that this method can be used to identify in which metabolic pathways the unannotated multigene families were involved, and consequently to gain deeper insights into their function.

## Discussion

LABs are used worldwide industrially in the manufacture of fermented foods and beverages (Holzapfel and Wood 2014), because their metabolic products improve nutritional value, organoleptic properties, as well as the microbiological safety of food products (Thierry et al. 2015). Since LAB use preferably carbohydrates as the primary carbon and energy sources, understanding the genetic basis of their utilization in metabolic pathways is essential to optimize the fermentative processes.

In this study, the comparison of LAB genomes revealed 219 single-copy genes shared among the 56 strains, which is known as the core genome. In another study comparing 20 *Lactobacillus* genomes, the core gene set was estimated to include 383 genes (Kant et al. 2011). Increasing the number of genomes to 213 reduced the core gene set to 73 genes (Sun et al. 2015). The core gene set is not only reduced with the number of compared genomes but also with the phylogenetic distances between them and the incompleteness of genome assemblies. For instance, Lukjancenko and colleagues (Lukjancenko et al. 2012) compared 81 LAB genomes from 6 different genera, and found that the core genome contains 63 genes. A greater core gene set of 172 single-copy genes was estimated in another study including 174 type strains from only 2 genera, *Lactobacillus* and *Pediococcus* (Zheng et al. 2015). In this study, the core gene set of 219 orthologous groups was slightly greater than those estimated in previous studies, probably reflecting that all genomes investigated were assembled at least at the scaffold level, precisely to improve gene completeness.

Functional annotation revealed that these 219 genes are mostly involved in nitrogen metabolism (data not shown). This suggests that these core genes are essential. Single-copy genes were unlikely duplicated or lost during evolution, implying they are true orthologs. Therefore, they reflect the divergence and phylogenetic relationship between the LAB species. Leveraging these 219 core genes, the phylogenetic tree was constructed, rooted assuming *St*. *salivarius* ssp. *thermophilus* and *Lc*. *lactis* as outgroup species (Fig. 3). Early after the first genomic studies, *Lactobacillus* species were found to belong to two separate clades (Makarova et al. 2006; Makarova and Koonin 2007; O’Sullivan et al. 2009; Salvetti et al. 2018). Our maximum-likelihood (ML) phylogenetic reconstruction recovered these two major *Lactobacillus* clades, referred as clade B and C, with high bootstrap support (Fig. 3). Nevertheless, the species that comprised these two clades have been traditionally controversial. The maximum phylogenetic uncertainty is associated with the species of the *Lb*. *casei* group, which harbor a large diversity in gene content across strains, including horizontal gene transfer (HGT) from *Lactobacilli* species of clade B (Broadbent et al. 2012). Indeed, multiple studies placed *Lb*. *casei* species within clade C (Makarova et al. 2006; Makarova and Koonin 2007; Claesson et al. 2008; Zhang et al. 2011; Salvetti et al. 2013; Sun et al. 2015), whereas a few others within clade B (Liu et al. 2008; O’Sullivan et al. 2009; Lukjancenko et al. 2012; Zheng et al. 2015). Our reconstruction based on a greater number of core genes supports the former hypothesis, embedding the *Lb*. *casei* group within group C (Fig. 3). Our phylogenomic tree also support *Leuconostoc* and *Pediococcus* as emerging within the *Lactobacillus* species of clade B, confirming previous results (Makarova et al. 2006; Makarova and Koonin 2007; Claesson et al. 2008; Liu et al. 2008; Zhang et al. 2011; Salvetti et al. 2013; Sun et al. 2015).

Mapping the fermentative types of 56 LAB revealed that some phylogenetically related species have different fermentation capabilities, a phenomenon that occurred within both, clades B and C, and that has been previously reported (Hammes and Vogel 1995; Felis and Dellaglio 2007; Pot et al. 2014), but disregarded by Zheng and colleagues (Zheng et al. 2015) in a more recent study. This incongruence could be explained by misclassifying the fermentation capabilities of a very few *Lactobacillus* strains. For example, *Lb*. *vini* has been assigned as a homofermentative species (Zheng et al. 2015), despite it produces small amounts of ethanol from arabinose and ribose (Felis and Dellaglio 2007; Endo and Dicks 2014), which were indeed degraded by *Lb*. *vini* in this study (Fig. 4).

A similar approach has been recently adopted to map lifestyle transitions (from free-living to host-adapted) onto certain phylogenetic nodes (Duar et al. 2017). Interestingly, the phylogenetic clades in this study mirror the grouping of these lifestyles. More specifically, most obligately heterofermentative strains of phylogenetic clade B, such as *Lb*. *brevis* and *Lb*. *parakefiri*, were classified as free-living (Duar et al. 2017). In contrast, all homofermentative strains of clade C, and for which lifestyle information is available, were classified as adapted to vertebrates (Fig. 3). Taken together, this suggests that fermentation types and lifestyles strongly shaped LAB genomic evolution. For example, and with the most notable exception of the *Lb*. *delbrueckii* group, GC content tends to be lower in homofermentative species (Fig. 3), most of which are host-adapted bacteria (Duar et al. 2017). Drastic variation in GC content is usually associated with gene acquisition through HGT (Garcia-Vallvé et al. 2000; Guindon and Perrière 2001), suggesting that heterofermentative capabilities could have been acquired from phylogenetically distant species. Regardless of the mechanism of gene acquisition, the gene repertoire of *Lactobacilalles* species is known to be highly dynamic (Zheng et al. 2015; Papizadeh et al. 2017), as reflected by the limited number of core genes found in this and other studies (O’Sullivan et al. 2009; Kant et al. 2011; Lukjancenko et al. 2012). Therefore, most of the LAB phenotypic variation is expected to result from gene gain and loss processes. In *Lactobacillus*, some genes related to carbohydrate utilization, for example, have been inferred to be acquired by HGT (Barrangou et al. 2003; Klaenhammer et al. 2005). Likewise, Zheng and colleagues (Zheng et al. 2015) found that genes lost in heterofermentative species are mostly associated with carbohydrate metabolism. Collectively, this supports gene variation as a major source of functional innovation in carbohydrate metabolism, and justifies genotype-phenotype association studies at the orthogroup level. Phenotyping different features of the carbohydrate metabolism revealed that LAB are separated in three metabolic clusters (Fig. 4). These clusters do not fully recapitulate the exact phylogenetic clades. This implies that some of these metabolic traits evolved along the diversification of the 56 LAB species, but others might have been acquired through HGT or independently lost in multiple lineages. Interestingly, the presence of certain genes not always implies degradation of the associated carbohydrate. For example, *St*. *thermophilus* was not able to metabolize salicin despite harboring multiple genes that in the other species were associated with salicin degradation (e.g., OG0000017, in Figs. 4 and 5). This is in line with *St*. *thermophilus* undergoing a massive pseudogenization process (gene inactivation), which is also observed in *Lb*. *helveticus* and *Lb*. *delbrueckii* (Bolotin et al. 2004; O’Sullivan et al. 2009). Conversely, *Lb*. *sanfranciscensis* was able to utilize diverse sources of carbohydrates, such as d-mannose (Fig. 4), albeit it lacked all gene families found to be significantly associated with its degradation (Fig. 5). These findings emphasize that phenotypes cannot be always inferred from the presence/absence of specific genes, but require explicitly integrating phenotypic information. Yet, this study revealed another interesting pattern. Metabolic cluster 3 contained all obligately heterofermentative strains (Fig. 5), and the association results indicated that all members within this cluster lacked 1-phospofructokinase (PFK), in contrast to the other two metabolic clusters, where this gene was present at least as single-copy (Fig. 5). PFK was found to be a key gene distinguishing hetero- and homofermentative species in previous studies (Morita et al. 2008; Zheng et al. 2015), and more recently confirmed based on phylogenetic framework (Salvetti et al. 2018). The association study implemented in this work provides further insights, revealing that PFK is indispensable not only for mediating fructose-6-P phosphorylation but also for d-mannose degradation during homofermentation. Since PFK acts on the homofermentative pathway, species lacking this enzyme produce CO_2_, ethanol, and lactate, through the heterofermentative pathway. Besides the PFK absence, obligately but also facultative heterofermentative species are partially characterized by the presence of three genes, represented by OG0001662, OG0001748, and OG0001480 (Fig. 5). These genes are associated with the degradation of l-arabinose, and annotated as l-arabinose isomerase, l-ribulose kinase, and ribulose phosphate epimerase, respectively. BLAST homology searches suggested that these genes correspond to *araA*, *araB*, and *araD*, conforming the *araBAD* operon. In *Escherichia coli*, this operon has been described to degrade l-arabinose into xylulose-5P, an essential compound in the heterofermentative pathway (Schleif 2010). Therefore, obligately heterofermentative strains are characterized by the absence of 1-phospofructokinase, and to a lesser extent by the presence of the three enzymes forming the *araBAD* operon (Fig. 5).

Metabolic clusters 1 and 2 excluded obligately heterofermentative strains, and only contained homofermentative and facultative heterofermentative LAB (Fig. 5). Particularly, cluster 1 showed an increased number of genes annotated as (i) carbohydrate phosphotransferase system (PTS); (ii) transcriptional regulators, such as the RpiR and DeoR families, and a transcription antiterminator; and (iii) glycosyl hydrolases, namely 6-phospho-β-glucosidase and sucrose-6-phosphate hydrolase. Interestingly, all these genes participate in the uptake of different carbohydrates, and their subsequent hydrolysis. PTS proteins are known to first import and phosphorylate carbohydrate substrates, such as salicin, d-cellobiose, and d-mannose. According to the results of this study, their degradation is significantly associated with the number of PTS transporters (Fig. 5). This is in line with other studies that have also described variation in PTS abundance across species, depending on their fermentation capabilities. More specifically, PTS components have been found to be numerous in homofermentative strains, while rarely in heterofermentative strains (Reizer et al. 1988; Bolotin et al. 2001; Michlmayr and Kneifel 2014). Despite being rare, PTS components had been described in only two obligately heterofermentative species, namely *Lb*. *brevis* (Saier et al. 1996) and *Lb*. *fermentum* (Zhang et al. 2013). This study corroborates PTS presence in *Lb*. *brevis* and *Lb*. *fermentum*, but also expands this observation to the type strains of the following obligately heterofermentative species: *Lb*. *buchneri*, *Lb*. *parakefiri*, *Lb*. *kefiri*, *Lb*. *hilgardii*, *Lb*. *malefermentans*, and *Lb*. *reuteri* (Fig. 5).

Following carbohydrate uptake by PTS proteins, transcriptional regulators modulate the degradation of β-glucosides like d-cellobiose, as well as aromatic β-glucosides, such as salicin and arbutin (Bardowski et al. 1994; Sonowal et al. 2013). All three carbohydrates, d-cellobiose, salicin, and arbutin, were shown in this study to be significantly associated with transcriptional regulators, supporting their role in the degradation of these β-glucosides (Fig. 5). Finally, glycosyl hydrolases are known to be also involved in the breakdown of β-glucosides in the cytoplasm. This association was also reflected in our results, as two glycosyl hydrolases were associated with the metabolism of d-mannose, salicin, and d-saccharose (Fig. 5). Interestingly, obligately heterofermentative strains within metabolic cluster 3 are often devoid of these two glycosyl hydrolases (Fig. 5), in line with previous studies finding a lower amount of glycosyl hydrolases in obligately heterofermentative *Lactobacillus* species (Michlmayr et al. 2013). More interestingly, PTSs, transcriptional regulators, and glycosyl hydrolases are often arranged in gene clusters, suggesting they orchestrate the co-regulation of carbohydrate metabolism (Morita et al. 2009), as potentially reflected by the results of the present study found for cluster 1 (Fig. 5).

In conclusion, genotype-phenotype association enabled the identification of genes involved and responsible for the transport and metabolization of specific carbon sources. Functional annotation of those orthogroups shed light into the metabolic pathways underlying different fermentative capabilities of selected LAB strains. As an example, PFK absence in obligately heterofermentative strains mirrored its essential function in the homolactic fermentation pathway. Complementarily, the results of this study also revealed novel associations between unannotated orthogroups (i.e., hypothetical proteins) with essential traits of the LAB carbohydrate metabolism, including OG0002201 (involved in d-melezitose degradation) and OG0001154 (with arbutin, salicin, and d-cellobiose). This highlights how genotype-phenotype associations can provide deeper insights into the function of hypothetical proteins. Further validation is required to confirm these findings, as well as to elucidate the potential roles of these genes in carbohydrate metabolism, which is of paramount importance for industrial applications.

## Electronic supplementary material

ESM 1 (PDF 207 kb)

ESM 2 (XLSX 89 kb)
