# Supplementary material for: Uncovering carbohydrate metabolism through a genotype-phenotype association study of 56 lactic acid bacteria genomes
Source: Appl Microbiol Biotechnol. 2019 Mar 4;103(7):3135–52. doi: 10.1007/s00253-019-09701-6 (PMC6447522; doi:10.1007/s00253-019-09701-6)
Supplement: Supplementary file 1 — (PDF 207 kb) [file 253_2019_9701_MOESM1_ESM.pdf]

Supplemental material

Journal: Applied Microbiology and Biotechnology

Title: Uncovering carbohydrate metabolism through a genotype-phenotype association study of 56 lactic acid bacteria genomes

Authors: Gemma Buron-Moles[a]*, Anna Chailyan[a], Igor Dolejs[a], Jochen Forster[a]*, Marta Hanna Mikš[a,b,c]

Author's affiliation(s) and address(es):

[a] Carlsberg Research Laboratory, J.C. Jacobsens Gade 4, 1799 Copenhagen V., Denmark

[b] Faculty of Food Science, University of Warmia and Mazury, Plac Cieszyński 1, 10-726 Olsztyn, Poland

[c] Glycom A/S, Kogle Allé 4, 2970 Hørsholm, Denmark

*Corresponding authors information:

Name: Jochen Forster / Gemma Buron-Moles

E-mail: jochen.forster@carlsberg.com / gemmaburonmoles@gmail.com

Telephone number: +45 4179 4132

Fax number: +45 3327 4700

Table S1 Assembly and genome statistics for 18 LAB species sequenced de novo in this work, in comparison to the genomes assembly that were previously available at NCBI.

				**Before (NCBI data 15/04/2018)**					**After (this work)**					**Fold change***	
**#**	**Taxon**	**Strain codes**		**# contigs**	**N50 (bp)**	**# proteins**	**%GC**	**Mb**	**# contigs**	**N50 (bp)**	**# proteins**	**%GC**	**Mb**	**# contigs**	**N50**
7	*Streptococcus salivarius* ssp. *thermophilus*	DSM 20617	ATCC 19258	NA	NA	NA	NA	NA	83	55173	2426	38.9	2.07	NA	NA
8	*Pediococcus pentosaceus*	DSM 20336		28	295919	1619	37.3	1.74	18	302347	1715	37.3	1.76	1.6	1.02
9	*Lactococcus lactis* ssp. *lactis*	DSM 20481	ATCC 19435	171	105989	2373	35.3	2.55	78	161786	2680	35.4	2.63	2.2	1.53
10	*Lactococcus lactis* ssp. *cremoris*	DSM 20069	ATCC 19257	213	17351	2030	35.6	2.27	208	20249	2950	36.0	2.57	1.0	1.17
11	*Lactobacillus sakei* ssp. *sakei*	DSM 20017	ATCC 15521	37	143342	1634	41.1	1.91	27	240687	1929	41.1	1.94	1.4	1.68
12	*Lactobacillus amylolyticus*	DSM 11664		73	49688	1403	38.3	1.54	52	58663	1680	38.4	1.63	1.4	1.18
13	*Lactobacillus delbrueckii* ssp. *jakobsenii*	DSM 26046		135	56583	1566	50.3	1.75	63	74016	1787	50.1	1.78	2.1	1.31
14	*Leuconostoc citreum*	DSM 5577	ATCC 49370	NA	NA	NA	NA	NA	10	522104	1802	39.0	1.84	NA	NA
15	*Leuconostoc fallax*	DSM 20189		30	107946	1356	37.5	1.64	16	273258	1648	37.7	1.66	1.9	2.53
16	*Lactobacillus silagei*	DSM 27022		88	170102	2372	44.9	2.67	34	157362	2518	45.0	2.71	2.6	0.93
17	*Lactobacillus paracasei* ssp. *paracasei*	DSM 5622	ATCC 25302	170	57042	2781	46.5	2.88	125	56289	3105	46.5	3.00	1.4	0.99
18	*Lactobacillus parakefiri*	DSM 10551		506	33253	4565	42.6	4.87	101	50705	2702	43.6	2.54	5.0	1.52
19	*Lactobacillus pentosus*	DSM 20314	ATCC 8041	152	48077	3122	46.3	3.65	42	188995	3366	46.3	3.70	3.6	3.93
20	*Lactobacillus farciminis*	DSM 20184	ATCC 29644	76	112984	2352	36.4	2.48	29	175159	2483	36.4	2.53	2.6	1.55
21	*Lactobacillus malefermentans*	DSM 5705	ATCC 49373	152	37650	1947	41	2.05	109	36706	2268	41.0	2.22	1.4	0.97
22	*Lactobacillus buchneri*	DSM 20057	ATCC 4005	90	64181	2228	44.4	2.45	74	65569	2482	44.4	2.55	1.2	1.02
23	*Lactobacillus pasteurii*	DSM 23907		30	170009	1714	38.6	1.91	22	312889	1816	38.6	1.90	1.4	1.84
24	*Lactobacillus hilgardii*	DSM 20176	ATCC 8290	125	64046	2379	39.6	2.60	92	64589	2732	39.9	2.77	1.4	1.01

*Note the reduction of the number of contigs and increased N50, as reflected in the last two columns.

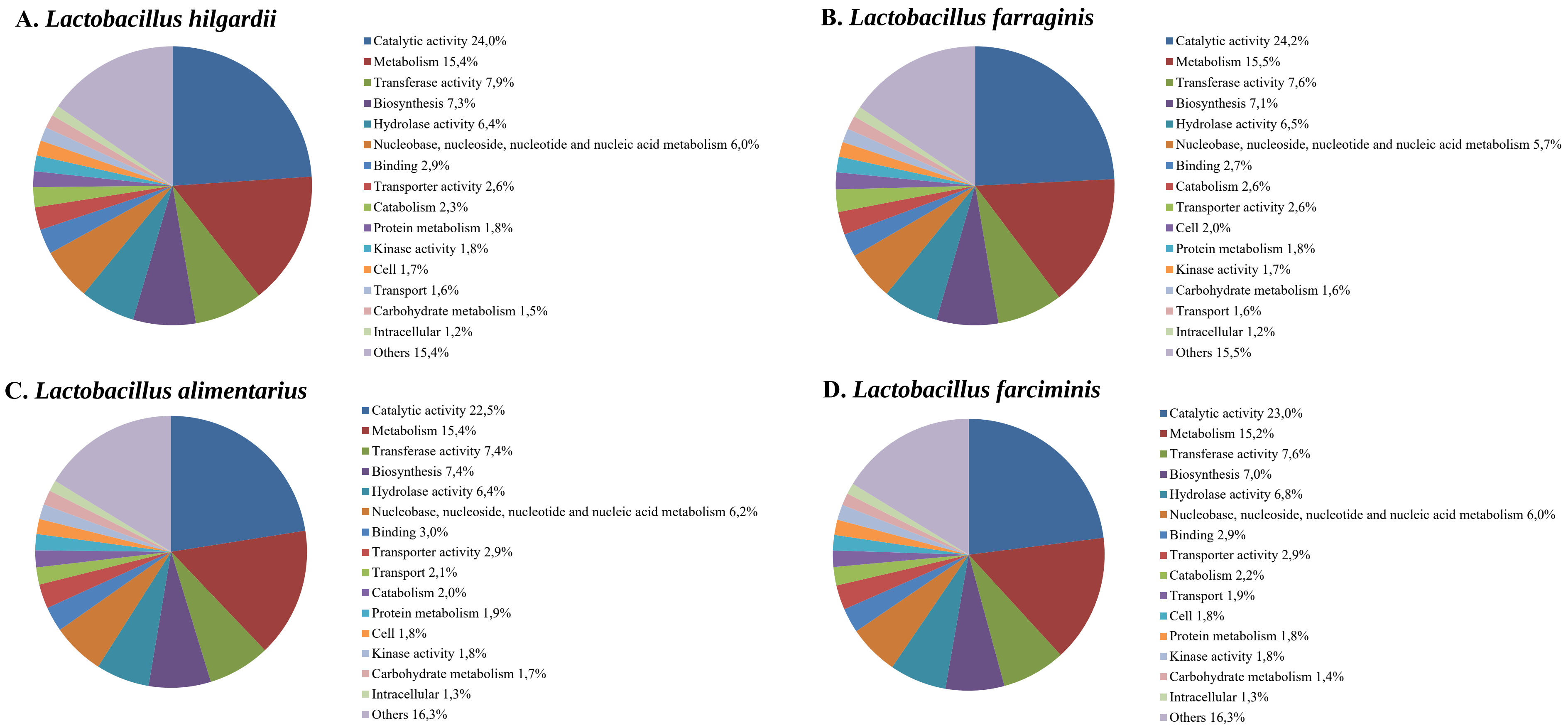

Fig. S1 Pie charts illustrating the GO terms distribution across four representative species, as calculated by the CateGOrizer software. Note the similar proportion of GO terms within each bacteria. GO categories in the corresponding legends are ranked decreasingly, based on their abundance in their respective pie chart. The numbers on each fraction indicate the percentage for each category.
